# Trastuzumab-Mediated Cardiotoxicity and Its Preventive Intervention by Zingerone through Antioxidant and Inflammatory Pathway in Rats

**DOI:** 10.3390/jpm13050750

**Published:** 2023-04-27

**Authors:** Gyas Khan, Mohammad Firoz Alam, Saeed Alshahrani, Yosif Almoshari, Abdulmajeed M. Jali, Saud Alqahtani, Mohammad Khalid, Shehla Nasar Mir Najib Ullah, Tarique Anwer

**Affiliations:** 1 Department of Pharmacology and Toxicology, College of Pharmacy, Jazan University, Jazan 45142, Saudi Arabia; 2 Department of Pharmaceutics, College of Pharmacy, Jazan University, Jazan 45142, Saudi Arabia; 3 Department of Pharmacology, College of Pharmacy, King Khalid University, Abha 61421, Saudi Arabia; 4 Department of Pharmacognosy, College of Pharmacy, Prince Sattam Bin Abdulaziz University, Alkharj 16278, Saudi Arabia; 5 Department of Pharmacognosy, College of Pharmacy, King Khalid University, Abha 61421, Saudi Arabia

**Keywords:** zingerone, trastuzumab, cardiotoxicity, interleukin, IL-2 and TNF-α, histopathology

## Abstract

Trastuzumab (TZB) is a new medicine, used to treat cancers of the breast and stomach. However, the cardiotoxic potential of this drug edges out its clinical advantages. The present study was designed to find out the effect of zingerone against trastuzumab-mediated cardiotoxicity in rats. In this study, five groups of rats with eight animals in each group were used. Group 1 was treated with normal saline, as a normal control (NC); Group 2 was treated with TZB (6 mg/kg/week-for five weeks) intraperitoneally as a toxic control. Groups 3 and 4 were pre-treated with zingerone (50 and 100 mg/kg, as per their body weight orally) along with five doses of TZB for five weeks, and Group 5 was treated with zingerone (100 mg/kg, body weight orally) as a control. TZB treatment showed cardiotoxicity as evidenced by increased levels of aspartate aminotransferase (AST), creatine kinase-myocardial band (CK-MB), lactate dehydrogenase (LDH), and lipid peroxidation (LPO) and decreased level of glutathione (GSH), and antioxidant enzymes such as glutathione peroxidase (GPx), glutathione reductase (GR), glutathione-s- transferase (GST), catalase (CAT), and superoxide dismutase (SOD) activities. Zingerone pre-treatment significantly decreased the levels of AST, CK-MB, LDH, and LPO and increased GSH and antioxidant enzymes content toward their normal level. In the TZB-alone administered group, inflammatory cytokines (IL-2 and TNF-α) levels were also elevated. Pre-treatment with zingerone restored the level of IL-2 and TNF-α toward normal level. The current findings undoubtedly demonstrated zingerone’s cardioprotective nature against TZB-mediated cardiotoxicity in rats with the evidence of histopathological recall.

## 1. Introduction

Cardiotoxicity is the most significant adverse effect of anticancer medications, leading to increases in disease and death in developed and developing nations [1,2]. The cardiotoxicity induced by several anti-neoplastic regimens on cardiac tissue may lead to myocarditis, myocardial infarction, congestive heart failure, and may even cause fibrosis of the myocardium [3]. In addition, the treatment pattern of anticancer regimens prefers the multidrug combination, which usually leads to serious synergistic adverse effects and has become the key cause for the withdrawal of such drug from the list of important drugs to treat cancer [4]. The use of drugs such as cyclophosphamide, doxorubicin, nivolumab, and trastuzumab can cause cardiac complications that may vary from myocardial abnormalities to irreversible heart failure [5,6]. Trastuzumab (TZB) is a humanized monoclonal antibody primarily used to treat human growth factor receptor-2 (HER2) positive breast cancer and stomach cancers [7]. HER2 linked signaling is important for development, survival, and inhibition of apoptosis of myocardial tissues. Whenever cardiomyocytes are exposed to biochemical or oxidative stress (hypoxia, concurrent use of antineoplastic drugs such as anthracyclines, cyclophosphamide, and trastuzumab etc.), neuroregulin binds to HER2/HER4 heterodimers, thereby promoting heart cell survival through the activation of phosphatidylinositide 3-kinase (PI3K) and mitogen activated protein kinase (MAPK) pathway [8,9]. TZB binds specifically and strongly to the extracellular domain IV of the HER2/neu protein found on cancer cells, thus inhibiting the activation of receptor tyrosine- protein kinase erbB-2/proto-oncogene neu (HER2/neu), which leads to inhibition of growth to specific breast cancer cells. Sufficient data have shown recently that HER2 also has an important role in embryonic heart development and in cardio-protection in the adult heart [10]. Furthermore, TZB administration triggers cellular oxidative stress and persuades the activation and expression of proapoptotic proteins. These circumstances all finally lead to mitochondrial impairment, subsequently causing the opening of mitochondrial permeability transition pore (MPTP) and the activation of cell death pathway, which accelerate myocardial dysfunction [11].

TZB after binding with cancerous cells inhibits their multiplication and stimulates the immune system abolishing cancerous cells [12]. This chemotherapeutic drug is used as an adjuvant in the treatment of metastatic cancer along with other anti-cancerous drugs. Trastuzumab is often a safe and effective treatment, although throughout clinical usage of this medication, several incidences of cardiotoxicity have been recorded [12,13]. Although the mechanisms of cardiotoxicity mediated by TZB are well understood clinically, relatively few studies on TZB-mediated cardiotoxicity in animal models have been conducted based on a mechanistic approach, motivating us to investigate natural active components as a therapeutically effective and safe cardioprotective against TZB.

To reduce the cardiotoxicity in cancer patients, numerous medicinal plants have been investigated; however, findings about these medications are still limited. Henceforth, natural plant active ingredients may become an important area of research to explore the cardioprotective effect against specific chemotherapeutic agents. Ginger (*Zingiber officinale*) is a herbaceous flowering plant whose rhizome is consumed throughout the world as an important spice and used as a condiment. In traditional oriental medicine (Ayurvedic Chinese, and Unani system of medicine), ginger has also been used for more than 2500 years to treat diseases such as rheumatoid arthritis, sore throat, nausea, constipation, indigestion, fever, infectious disease, and in the treatment of helminthiasis [14]. Moreover, as well as these important pharmacological properties, ginger is also used as an anti-inflammatory, an immune modulator, in tumor suppression, diabetes mellitus, hyperlipidemia, and many more diseases [15]. Many bioactive phenolic compounds have been isolated from ginger, including non-volatile pungent compounds such as gingerols, shogaols, zingerones, etc. Zingerone is the one of the active constituents of ginger, which has a phenolic alkanone [16,17]. Zingerone has anti-inflammatory, antioxidant, and anti-apoptotic properties and protects from radiation-mediated cytotoxicity [18,19,20]. In addition to this usefulness in the treatment of various disorders, the molecular mechanism of zingerone is not well understood against trastuzumab-mediated cardiotoxicity in rats. Therefore, this study was designed to understand the therapeutic action of zingerone and its molecular mechanism in respect to cardio-protection against TZB- mediated cardiotoxicity in rat.

## 2. Materials and Methods

### 2.1. Animals

Wistar rats (150–200 g) were chosen and housed in a well-ventilated animal room at Jazan University’s College of Pharmacy for one week prior to the commencement of the experimental design to allow for optimal acclimation under controlled conditions. Animals were provided ideal conditions along with regular food and water during the experimental period. This study was approved by the Institutional Research Review and Ethics Committee of Faculty of Pharmacy, Jazan University (approval number 814/706/1440).

### 2.2. Drugs and Chemicals

Trastuzumab (Herceptin 150 mg/mL) was acquired from Roche Pharma (Basel, Switzerland), while zingerone was obtained from Sigma Aldrich, Co, Germany, via Bayoni trade firm of Saudi Arabia. Elisa Assay Kits for inflammatory cytokines IL-2 and tumor necrosis factor alpha (TNFα), lactate dehydrogenase (LDH), aspartate aminotransferase (AST), and creatine kinase-mayocardial band (CK-MB) were purchased from Abcam, United Kingdom in Saudi Arabia via a local supplier.

### 2.3. Experimental Protocol

Forty rats were distributed into five groups. Group 1 acted as the standard control and received normal saline. Group 2 served as a toxic control and administered intraperitoneal TZB (6 mg/kg, bw/week) to induce cardiotoxicity [21]. Groups 3 and 4 got oral dosage of zingerone (50 and 100 mg/kg, bw, respectively) and intraperitoneal injection of TZB (6 mg/kg/week). Positive control Group 5 got an oral dose of 100 mg/kg of zingerone. Five weeks of the above therapy continued (35 days).

### 2.4. Dissection and Homogenization

After five weeks, animals were euthanized under the influence of anesthesia (ketamine (100 mg/mL) and xylazine (7 mg/kg)) and serum was taken for biochemical analysis. Each rat’s heart was immediately removed, cleaned with normal saline, and homogenized at 40 degrees Celsius in phosphate buffer (0.1 M, pH 7.4) with KCl (1.17% *w*/*w*). At 4 °C, the homogenized tissue was centrifuged again to get post mitochondrial supernatant (PMS).

### 2.5. Biochemical Estimations

#### 2.5.1. Determination of AST

Reitman and Frankel’s [22] approach was used to calculate AST. Aspartate transaminase catalyzes the amino group transfer from L-aspartate to α-ketoglutarate, culminating in the production of oxaloacetate and glutamate. Oxaloacetate interacts with 2,4-dinitrophenylhydrazine (DNPH) to form a brown-colored 2,4-dinitrophenylhydrazone derivative in alkaline media. At 505 nm, the absorbance was measured using a colorimeter.

#### 2.5.2. Determination of CK

David and Rovert’s approach [23] was used to calculate creatine kinase activity. In brief, the excess creatine kinase catalyzes the conversion of creatine phosphate and ADP into ATP. Hexokinase converts glucose to glucose-6-phosphate and it further oxidized to 6-phosphategluconate by glucose-6-phosphate dehydrogenase using ATP, which reduces NADP+ to NADPH. The absorbance at 340 nm is directly proportional to CK.

#### 2.5.3. LDH Assay

LDH assay was performed as per Lum and Gambino protocol [24]. LDH catalyzes the lactate to pyruvate by oxidation while simultaneously reducing NAD+ to NADH+ H+. The quantity of pyruvate produced was measured at 340 nm. The serum LDH activity is proportional to the rise in absorbance resulting from the depletion of NAD+.

#### 2.5.4. LPO Assay

LPO assay was determined by Ohkawa et al. where thiobarbituric acid (TBA) reacts with malondialdehyde (MDA) which is formed as a result of peroxidation of membrane lipids, which help to calculate LPO in term of TBARS (Thiobarbituric acid reactive substance) [25]. In brief, 10% homogenized tissues were used for centrifuge at 10,500 g for the collection of supernatant. Further, 1 mL of supernatant was added with 30% TCA (0.5 mL), 0.8% TBA (0.5 mL) and the mixture was kept for 30 min at 80 °C in a water bath shaker. After 30 min, the tubes were removed and placed in chilled water for 10 min. It was further centrifuged for 15 min at 800 g. At room temperature, the absorbance of supernatant was measured at 540 nm. LPO was measured in milligrams of protein as nmoles of TBARS. The protein content of diverse samples was calculated by using the Lowry technique [26]. 

#### 2.5.5. GSH Assay

GSH content was calculated using the Sedlak and Lindsay [27] technique. In 0.02 M EDTA, the tissue was homogenized. In a test tube, 5 mL homogenates were combined with 50% TCA (1 mL) and 4 mL of chilled distilled water. The tubes were mixed with the help of vortex mixer for 10 min and centrifuged for 15 min at 1200× g. Further, 2 mL of supernatant was combined with 4 mL of Tris-buffer (0.4 M, pH 8.9) and after mixing the solution, 0.1 mL of 0.01 M DTNB (5,5′-dithio-bis (2-nitro benzoic acid)) was added and absorbance was taken within five minutes of addition of DTNB at 412 nm by using UV-spectrophotometer against a reagent blank (without any homogenate).

#### 2.5.6. GPx Assay

GPx activity was assessed by using the procedure of Mohandas [28]. In this method, the total 2 mL volume of the reaction mixture consisted of phosphate buffer (1.49 mL, 0.1 M, pH 7. 4), EDTA (0.1 mL, 1 mM), sodium azide (0.1 mL, 1 M), glutathione reductase (0.05 mL, 1 IU/mL), reduced glutathione (0.05 mL, 1 mM), NADPH (0.1 mL, 0.2 mM), H_2_O_2_ (0.01 mL, 0.25 mM), and at last 0.1 mL of post mitochondrial solution (10% *w*/*v*) was added to the reaction mixture. The NADPH disappearance was observed at 340 nm, at 25 degrees Celsius. The enzyme activity was calculated as nmol NADPH- oxidized per minute of per mg protein, using molar extinction coefficient of 6.22 × 103 M^−1^, cm^−1^. 

#### 2.5.7. GR Assay

The GR activity was assessed using Mohandas’s [28] technique. In brief, 2 mL of reaction mixture contained 1.65 mL phosphate buffer (0.1 M, pH 7.4), 0.1 mL of EDTA (0.5 nM), 0.05 mL of oxidized glutathione (1 mM), 0.1 mL of NADPH (0.1 mM), and 0.1 mL of PMS (10% *w*/*v*). The loss of NADPH at 340 nm was observed. The NADPH disappearance was observed at 340 nm, at 25 degrees Celsius. The enzyme activity was calculated as nmol NADPH- oxidized per minute of per mg protein, using molar extinction coefficient of 6.22 × 103 M^−1^, cm^−1^. 

#### 2.5.8. GST Assay

The Haque approach [29] was used to find the GST activity. In brief, 2 mL of reaction mixture was contained 1.675 mL phosphate buffer, 0.2 mL reduced glutathione, 0.025 mL 1-chloro, 2,4-dinitro benzene (CDNB), and 0.1 mL of 10 % *w*/*v* PMS. The GST estimated as nmol CDNB conjugates formed/min/mg protein using an absorbance of 340 nm, by using 9.6 × 103 M^−1^, cm^−1^ of molar extinction coefficient. 

#### 2.5.9. SOD Assay

SOD assay was determined using the Marklund and Marklund technique at 580 nm wavelength [30]. The enzyme activity is expressed in units per mg protein, with 1 unit of enzyme activity equaling 50% suppression of pyrogallol autoxidation.

#### 2.5.10. CAT Assay

Claiborne’s [31] technique was applied to estimate the activity of CAT. In brief, 3 mL volume contained 1.95 mL phosphate buffer, 1.0 mL H_2_O_2_, and 0.05 mL PMS. At 240 nm, absorbance changes were measured. The CAT was measured in nmoles of hydrogen peroxide consumed/min/mg of protein.

#### 2.5.11. Inflammatory Cytokine (IL-2 and TNF-α) Assay

ELISA Assay kits for inflammatory cytokine networks were established in cardiac tissue supernatant by using the Sandwich ELISA method. The standard calibration curve was drawn and the sample was measured according to the Abcam kit’s usual protocol. The generation of complex color was evaluated through the ELISA reader at 450 nm. The concentration of sample was calculated by using a standard curve.

#### 2.5.12. Histopathological Examination

The isolated heart was properly washed in chilled normal saline before being stored in a 10% buffered neutral formalin solution. The cardiac tissue was then embedded in paraffin [32] after being fixed. Afterwards, hematoxylin and eosin were used to stain sections of heart tissue (H&E). Additionally, photomicrographs were made after careful inspection under a high-magnification microscope (400×). Myocardial damage caused by trastuzumab was given a score between 0 and 4 based on the percentage of vacuolization inflammation, and myofibrillar loss in randomly chosen heart regions.

#### 2.5.13. Statistical Analysis

Means and standard deviations were calculated after analyzing the data statistically using the analysis of variance (ANOVA-one way) and Tukey-Kramer test. The significance threshold used was the *p* = 0.05 cutoff.

## 3. Results

### 3.1. Action of Zingerone on Serum Marker

Table 1 represents the zingerone action on serum markers (AST, CK-MB, and LDH). TZB administration (Group 2) caused significant (*p* < 0.001) enhancement of serum markers in comparison to normal control (Group 1). Zingerone pre-treated groups (3 and 4) indicated significant (*p* < 0.001) decrease in serum marker enzymes in comparison to TZB treated Group 2. Zingerone-alone-treated Group 5 indicated no change in the serum markers as compared to Group 1.

### 3.2. Action of Zingerone on TBARS

**Figure 1:**
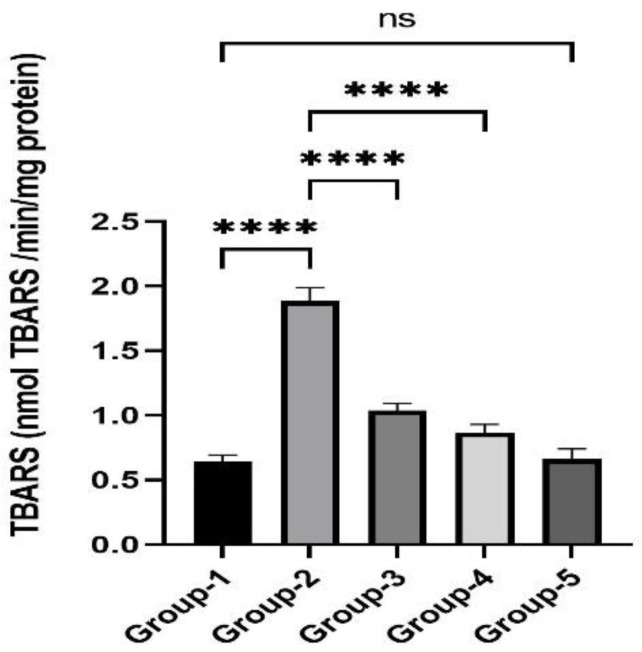
Effects of zingerone on thiobarbituric acid reactive substances (TBARS) after TZB-mediated cardiotoxicity. Data are represented as mean ± S.D (*n* = 8 rats). **** *p* < 0.05 Group 2 vs. Group 1, **** *p* < 0.01 vs. Group 3 and 4 vs. Group 2, ^ns^ *p* > 0.05 Group 5 vs. Group 1.

The impact of zingerone on TBARS is given in Figure 1. TZB administration resulted in a substantial (*p* < 0.001) rise in TBARS level in Group 2 as compared to Group 1. It was also noticed that after treatment with zingerone in Group 3 and Group 4, significantly, thiobarbituric acid reactive substances (TBARS) level was reduced as compared to Group 2. Group 5, which was treated with only zingerone, did not show any remarkable changes which indicated that there is no self toxicity of zingerone at the dose of 100 mg/kg body weight.

### 3.3. Action of Zingerone on Antioxidant

**Figure 2:**
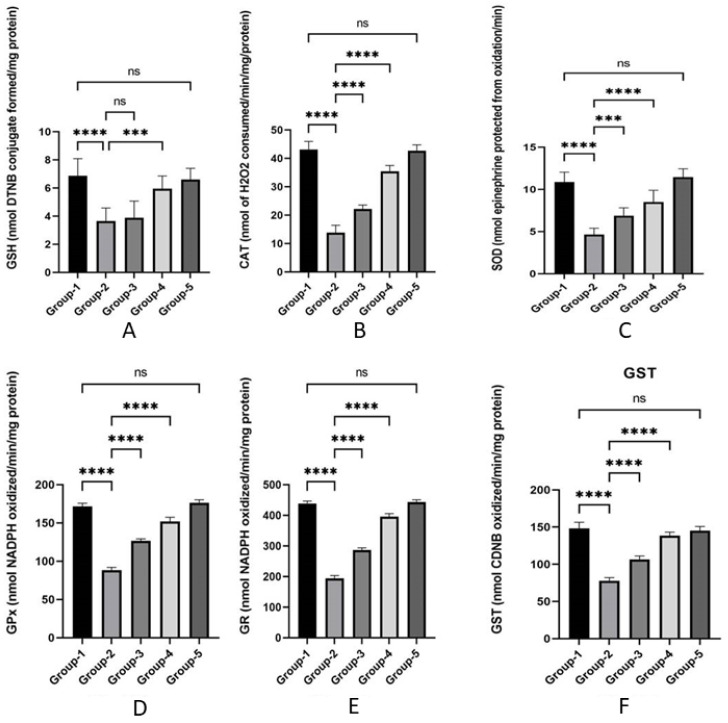
(**A**–**F**) Effects of zingerone effect on antioxidant enzymes after TZB-mediated cardiotoxicity. Data are represented as mean ± SD (*n* = 8 rats). **** *p* < 0.001 vs. Group 1, Group 2, Group 3, and Group 4; *** *p* < 0.001 vs Group 3, and Group 4; ns = non-significant (^ns^ *p* > 0.05) vs. Group 1.

The impact of zingerone on oxidative stress indicators (GSH, GPx, GR, GST, CAT, and SOD) are indicated in Figure 2A–F. TZB reduced the contents of GSH in Group 2 as compared to Group 1. Similar decreases in antioxidant enzyme levels (GPx, GR, GST, CAT, and SOD) were seen in Group 2 after TZB treatment compared to Group 1. Zingerone-treated Groups 3 and 4 substantially (*p* < 0.001) lowered LPO level, raised the level of non-enzymatic antioxidant (GSH), and restored enzymatic antioxidant (GPx, GR, GST, CAT, and SOD) to near normal level when compared with Group 2. No significant change was seen in oxidative stress indicators in the zingerone-only-treated Group 5 in comparison to normal control (Group 1).

### 3.4. Action of Zingerone on Inflammatory Cytokines

**Figure 3:**
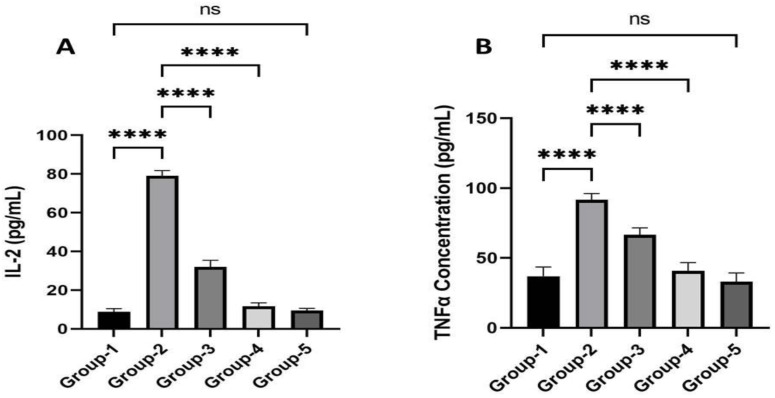
(**A**,**B**) represent the zingerone effects on cytokines. (**A**) represents its effects on IL-2 against trastuzumab-mediated cardiotoxicity in rats. **** *p* < 0.05 Group 2 vs. Group 1, **** *p* < 0.01 vs. Group 3 and 4 vs. Group 2, ^ns^ *p* > 0.05 Group 5 vs. Group 1. (**B**) represents its effect on TNF-α against trastuzumab-mediated cardiotoxicity in rats. **** *p* < 0.05 Group 2 vs. Group 1, **** *p* < 0.01 vs. Group 3 and 4 vs. Group 2, ^ns^ *p* > 0.05 Group 5 vs. Group 1.

Figure 3A,B showed the zingerone effect on inflammatory cytokines. The administration of TZB resulted in a significant increase in IL-2 and TNF-α in Group 2 in comparison to Group 1. Pre-treatment with zingerone resulted in a significant decrease in inflammatory cytokines levels in Groups 3 and 4 comparison to Group 2. There was no significant difference reported in Group 5 as compared to Group 1.

### 3.5. Action of Zingerone on Histopathological Assay

**Figure 4:**
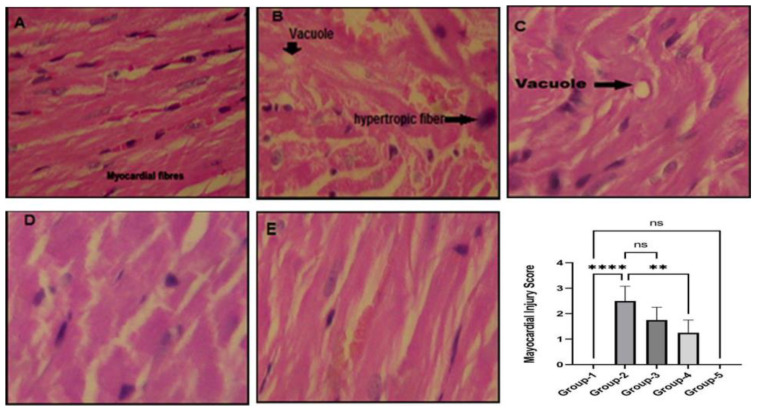
(**A**–**E**). Effect of Zingerone on histopathological alterations mediated by trastuzumab in rat’s heart. (**A**) represents normal control without any pathological changes, given myocardial injury score was 0; (**B**) TZB only treated group showing pathological changes with vacuolation and hypertropic fibers, given myocardial injury score was 2.5 which was significant **** *p* < 0.0001 vs. Group 1; (**C**) represents zingerone (50 mg)-treated rats with small vacuolation, where given myocardial injury score was 1.75 but it was not significant ^ns^ *p* > 0.05 vs. Group 2; (**D**) zingerone (100 mg)-treated rats with no vacuolation and significant restoration of myocardial fiber given myocardial injury score was 1.25, and it was significant ** *p* < 0.001 vs. Group 2; (**E**) represents zingerone-only treated group showing normal myocardial fiber injury score was 0 and it was not significant ^ns^ *p* > 0.05 vs. Group 2 (H & E X 400×).

Figure 4A–E, showed the effect of zingerone on histopathological changes in different group of animals. The administration of trastuzumab resulted in clear inflammation, vacuolation, necrosis with irregular shaped hypertrophic myocardial fiber in the rat heart in Group 2 when compared to Group 1. Rats pre-treated in Groups 3 and 4 with zingerone showed improvement in histopathological changes towards a normal shape of myocardial fiber. There was no inflammation, vacuolation, or necrosis found in Group 5 treated with the highest dose of zingerone.

## 4. Discussion

TZB is a commonly utilized biological medication for the HER2-positive breast cancer and gastric cancer. Unfortunately, regular uses of this drug resulted in development of cardiotoxicity such as myocarditis or congestive heart failure in cancer patient [33,34]. Therefore, it becomes an essential requirement to investigate the cardioprotective mechanism of natural products as compared to synthetic drugs. Zingerone is a natural active ingredient with various pharmacological action.

In our current study, the histopathological finding showed significant cellular disruptions such as inflammation of the sarcoplasmic reticulum, cytoplasmic vacuolization, and loss of myofibrils apoptosis with significant increase in pro-inflammatory cytokines (IL-2 and TNF-α) and serum cardiac marker enzymes (AST, CK-MB, and LDH) in TZB-treated Group 2. After treatment of zingerone in Groups 3 and 4, the cellular changes such as inflammation of sarcoplasmic reticulum, cytoplasmic vacuolization, and myofibril losses are restored successfully towards normal condition, compared to Group 2. In contrast, pro-inflammatory cytokines and cardiac serum marker enzymes levels were also restored towards normal level compared to Group 2 rats. 

Similarly, the morphometric estimation of cardiac tissues showed an increase in all indices suggesting oxidative stress, cellular inflammation, and apoptosis mediated by TZB. At the same time, quantitative estimation of most biochemical parameters, such as antioxidant enzymes, supported the histopathological findings. In our result, the levels of oxidant (GSH) and antioxidant enzymes (GPX, GR, GST, CAT, and SOD) significantly decreased in TZB-treated Group 2 compared with control Group 1. Similarly, the administration of TZB in Group 2 significantly elevated the LPO in the cardiac tissue as compared to Group 1. Treatment of zingerone in Group 3 and 4 rats improved cardiac biomarkers compared to TZB alone treated Group 2. Treatment of zingerone in Group 3 and 4 rats also increased the level of glutathione, as well as the level of antioxidant enzymes toward normal level compared to TZB alone treated Group 2. Indeed, previous studies also reported that administration of TZB and other anticancer drugs such as doxorubicin causes histopathological changes in the heart muscles, which concur with our results. In brief, African vegetables effectively mitigate TZB-mediated cardiotoxicity in rats Wistar rats [35]. Similarly, the cardio-protective effect of dexrazoxane on animal cardiotoxicity model mediated by anthracycline combine with trastuzumab is associated with upregulation of calpain-2. Yavas et al. highlighted that Spironolactone ameliorates the cardiovascular toxicity mediated by concomitant trastuzumab and thoracic radiotherapy. Recently, we reported that Thymoquinone ameliorates doxorubicin-mediated cardiotoxicity in Swiss albino rats by modulating oxidative damage and cellular inflammation [36,37,38,39]. 

In addition to its prominence in malignant cells, HER2 is also expressed in normal cardiomyocytes with its other family members (HER1, HER2, and HER4) [40]. HER2 along with its ligands Neuregulin-1 (NRG1) maintain the physiological condition and development of cardiomyocytes. Under stress conditions, cardiac microvascular endothelial cells can release NRG1, which binds to HER4 [41,42]. This cellular event triggers HER4/HER4 homo-dimerization or HER4/HER2 hetero-dimerization, which can further activate a series of pathways (MAPK, PI3K-AKt) and protect the cardiomyocytes from reactive oxygen species (ROS) and nitric oxide (NO) production [43]. When a patient is treated with trastuzumab, it binds to HER2 and inhibits the dimerization of HER4/HER2. This interferes with the protective pathway described above, which causes ROS production, the suppression of autophagy in the myocardium, the destruction of mitochondria, and the release of TNF-α expression, which leads to cardiotoxicity [44,45,46]. Some other studies have also supported our finding that TZB administration causes IL-2 and TNF-α enhancement in serum levels, which is responsible for cardiotoxicity [47,48].

It has been reported by Kang et al. (2011) that the myocardium is highly susceptible to free radicals, because the heart is equipped with a low content of antioxidant and having high affinity to ROS [49]. The elevated ROS in myocardial tissue led to cell membrane impairment due to lipid peroxidation and finally damaging the myocardial tissues [50,51]. Glutathione protects our cells from the harmful effects of ROS. A significant diminution in GSH content may augment the production of ROS, mitochondrial dysfunction, and decreased in ATP formation, which may lead to cell injury and finally cell death [52]. In this condition of GSH deficiency, the heart defense system provokes a compensatory response from other antioxidants such as GPX, GR, GST, CAT, and SOD to prevent the harmful effects of these unstable ionic species [53,54]. In this study, after TZB administration, the activity of these oxidant and antioxidant enzymes were markedly decreased, thus the free radical-quenching property of the heart is also decreased, resulting in oxidative stress and cardiotoxicity. 

Zingerone treatment reinstates the level of such oxidant and antioxidant enzymes toward normal levels and thus plays a key role in mitigating the cardiotoxicity induced by the administration of TZB. In addition to oxidative stress, the inflammatory cytokines pathway also shows a significant role in cardiotoxicity, and it was noticed in our study that TZB-treated animals exhibited a significant rise in IL-2 and TNF-α, which were further restored toward normal level as discussed above. Many other studies support our findings that TZB administration increases the production of inflammatory cytokines, and generation of reactive oxygen species as well as nitric oxide [55]. These inflammatory cytokine levels were managed by the zingerone treatment in a dose-dependent manner. 

Other studies have also supported our results, where TZB administration resulted in a significant increase in IL-2 and TNF-α level, which finally may cause cardiovascular complications. Moreover, elevated expression of pro-inflammatory cytokines such as IL-2 and TNF-α can induce cardiotoxicity and extended inflammatory reaction may become the reason for the development of cardiac fibrosis. Several studies reported the release of proinflammatory responses [56,57,58]. Similarly, the progression of cardiomyocytes damage leads to the destruction of cardiac cell membrane and thus serum markers enzymes such as AST, CK-MB, and LDH are leaked into the circulation [59,60]. Other researchers also reported the significant increase of cardiac enzymes after administration of TZB, which is evidence of TZB-indued cardiac toxicity. These cardiac biomarkers are reliable markers for diagnosing and monitoring cardiac disease. Very few studies have been reported for uses of antioxidants as adjuvant therapy to prevent cardiotoxicity. Recently, Mousa A. M. et al. worked on Allicin-mediated antioxidant adjuvant therapy used in an animal model to minimize the adverse effects of trastuzumab through antioxidant, anti-inflammatory, and antihyperlipidemic properties [21]. In this study, zingerone successfully attenuated the TZB-induced cardiotoxicity by enhancing the antioxidant system and reducing the inflammatory pathway in rats. Thus, zingerone can be suggested as an adjuvant therapy with TZB to minimize the impact of cardiotoxicity. This study uses an animal model and has a further need of exploration for humans.

## 5. Conclusions

In summary, the current investigation supports the cardioprotective action of zingerone by modifying serum markers, markers of oxidative stress, and inflammatory markers in TZB-mediated cardiotoxicity. The study corroborates the therapeutic potential of zingerone and suggests that it may be utilized in future to minimize the TZB-mediated cardiotoxicity.

## Figures and Tables

**Table 1:** Effect of zingerone on TZB-mediated cardiotoxicity markers in serum.

Groups	AST (mg/dL)	CK-MB (µ/L)	LDH (µ/L)
Group 1	60.93 ± 5.62	689.71 ± 4.51	490.66 ± 7.93
Group 2	265.12 ± 3.3 ^#^	3975.43 ± 8.32 ^#^	3395.57 ± 5.08 ^##^
Group 3	115.05 ± 3.10 *	3194.48 ± 6.97 *	1945.56 ± 5.38 **
Group 4	85.39 ± 5.57 ***	1790.97 ± 5.89 **	1465.41 ± 4.31 ***
Group 5	59.79 ± 6.03 ^ns^	695.99 ± 6.60 ^ns^	484.6 ± 4.08 ^ns^

Data are represented as mean ± SD (*n* = 8 rats). ^#^ *p* < 0.05, ^##^ *p* < 0.01 vs. Group 1. * *p* < 0.05, ** *p* < 0.01, *** *p* < 0.001 vs. Group 2. ns = non-significant (^ns^ *p* > 0.05) vs. Group 1.

## Data Availability

The data are given inside the text of the article.
